# *Nehiyawak* (Cree) women’s strategies for aging well: community-based participatory research in Maskwacîs, Alberta, Canada, by the *Sohkitehew* (Strong Heart) group

**DOI:** 10.1186/s12905-023-02453-6

**Published:** 2023-06-27

**Authors:** Luwana Listener, Sue Ross, Richard Oster, Bonny Graham, Seth Heckman, Cora Voyageur

**Affiliations:** 1 Department of Obstetrics and Gynecology, Faculty of Medicine and Dentistry, University of Alberta, Edmonton, AB Canada; 2 Indigenous Wellness Core, Alberta Health Services, Edmonton, AB Canada; 3 Maskwacîs Health Services, Maskwacîs, AB Canada; 4 Department of Sociology, Faculty of Arts, University of Calgary, Calgary, AB, Canada

**Keywords:** Indigenous women, Aging well, Wellness, Community based participatory research, Sharing circles, Medicine wheel, Community feedback

## Abstract

**Background:**

The *Sohkitehew* (Strong Heart) Research Group, which included an Elders Advisory Committee of seven *Nehiyawak* (Cree) women, set out to bring Maskwacîs community members together to understand *Nehiyawak* women’s experiences of “aging well”. The goals of this research were to generate information honouring Indigenous ways of knowing, and gather strengths-based knowledge about aging well, to help Maskwacîs, women maintain wellness as they age.

**Methods:**

We facilitated qualitative Sharing Circles in three different settings in Maskwacîs. Discussions were prompted using the four aspects of the self, guided by Medicine Wheel teachings: Physical, Mental, Emotional, Spiritual. Detailed notes were recorded on flip charts during the discussions of each Sharing Circle. Data were analysed using descriptive content analysis to identify practical strategies for aging well.

**Results:**

Thirty-six community members attended one or more Sharing Circle. Strategies included: Physical—keeping active to remain well; Mental—learning new skills to nourish your mind; Emotional—laughing, crying, and being happy; Spiritual—practicing *Nehiyawak* traditional ways. Participants commented that balancing these four aspects of the self is necessary to achieve wellness. Following the analysis of the Sharing Circle comments, three community feedback sessions were held to discuss the results in the wider community. These strategies were formatted into a draft booklet which incorporated Cree language, and archive photographs of Maskwacîs women and families.

**Conclusions:**

The *Nehiyawak* Sharing Circles identified practical strategies that help women to remain well as they age. This positive approach to aging could be adopted in other Indigenous and non-Indigenous communities.

## Background

### The role of *Nehiyawak* (Cree) women

Women’s traditional position in *Nehiyawak* communities is at the heart of the family, as the holders of traditional knowledge. Women give life to children, nurture older family members, while providing strength to entire communities. Many traditional *Nehiyawak* teachings and practices were damaged and dismantled by policies of *Cultural Genocide* (which sought to destroy First Nations^1^ society) and the impacts of *Colonization* (most notably government policy, intergenerational trauma and residential schools) that continue to be felt today [1, 2]. As a result of these past and continuing impacts, many opportunities for intergenerational teaching have been lost [3, 4]. Despite this history, women remain the core of *Nehiyawak* communities and women’s wellness is of vital importance to the whole community. Improving the wellness of *Nehiyawak* women is critical not only for individual women, but also for the revitalization of families and communities [5], ensuring that traditional teachings and practices are maintained and that families are fully supported. Women strive to remain healthy and well as they age – physically, mentally, emotionally, and spiritually—so they can support the healing and nurturing of family members, relatives and the community for the next seven generations [6].

### *Nehiyawak* peoples’ understanding of wellness

Wellness is a holistic term that aligns with *Nehiyawak* beliefs and worldview. This worldview is grounded in the belief that we are not just physical, but also mental, emotional and spiritual beings – core aspects of the Medicine Wheel [6]. As described by Elder Jim Dumont:

*Wellness from an Indigenous perspective is a whole and healthy person expressed through a sense of balance of body, mind, emotion, and spirit. Central to wellness is belief in one’s connection to language, land, beings of creation, and ancestry, supported by a caring family and environment* [7].

This widely acknowledged definition of holistic wellness highlights the importance of a comprehensive understanding that encompasses behaviours, beliefs, and understanding that help individuals and communities to create capacity, confidence, purpose, hope, identity, belonging, and meaning [8–10]. Dumont’s definition stresses the need for balance in the four components of the individual (physical, the mental, emotional and spiritual) indicating that wellness is much more than the absence of ill health. His definition guided the development of our research in Maskwacîs, Alberta, and the Medicine Wheel itself provided the framework for our data analysis.

### Setting for the research: the community of Maskwacîs

The *Nehiyawak* community of Maskwacîs is an unincorporated community in a rural setting located approximately 85 kms south of Edmonton, Alberta and situated between the City of Wetaskiwin and Town of Ponoka in the Treaty #6 region. The community consists of four *Nehiyawak* First Nations who share similar traditions, culture and language: Ermineskin Cree Nation, Louis Bull Tribe, Montana First Nation and Samson Cree First Nation. Each of these Nations is responsible for their own leadership and management. Together the four Nations form the Maskwacîs Tribal Council. Pigeon Lake is a community located 50 kms from Maskwacîs and includes members from all four Nations. The combined land mass of Maskwacîs and Pigeon Lake is approximately 310 square kilometres, mainly agricultural land. According to the 2021 Statistics Canada Census Profile of the communities in Maskwacîs, the combined population including all four Maskwacîs bands was 7,728, with around 33% of the population being 0 to 14 years of age, 62% aged 15 to 64 years, and 5% aged 65 years or older. https://www12.statcan.gc.ca/census-recensement/2021/dp-pd/prof/index.cfm?Lang=E.

Maskwacîs members are registered with, and received healthcare from, Maskwacîs Health Services (MHS) which operates health and wellness clinics in each Nation and in Pigeon Lake. Social support is also provided in each setting. Maskwacîs Education Schools Commission (MESC) includes 11 schools from kindergarten to secondary. Post-secondary education is available at Maskwacîs Cultural College (MCC). Each Nation and Pigeon Lake also have retail and commercial facilities, providing services and local employment opportunities. Other services and employment opportunities are available nearby in Wetaskiwin and Ponoka.

Originally developed to increase menopause awareness in the community [11], the *Sohkitehew* Research Group was formed in Maskwacîs in 2015 and included two women Elders, community members, health care staff, and researchers from the University of Alberta. When University of Calgary sociologist, Voyageur, joined to lead the Group, our goals evolved to encompass a more strengths-based approach to mature women’s health research with a focus on wellness. The *Sohkitehew* Elders Advisory Committee was formed in 2017 and included seven women Elders from the four Nations of Maskwacîs and Pigeon Lake. The Elders Advisory Committee became part of, and advises the *Sohkitehew* Research Group with traditional knowledge and best practices for engaging with the community in culturally responsive ways while providing guidance and support. The *Sohkitehew* Research Group, together with the Elders Advisory Committee, works with the wider Maskwacîs women’s community to explore, document, and disseminate information about ways to attain and maintain wellness as they age. 

### The purpose of this research

This preliminary research project was developed by the *Sohkitehew* Research Group to bring community members together to develop a mutual understanding about *Nehiyawak* women’s experiences of “aging well” by listening to each other’s comments and advice. The goals of the research project were:

- To generate information respecting and honouring Indigenous ways of knowing.
- To gather valuable strengths-based knowledge about aging well.
- To help other women in the community find their own cultural ways to attain and maintain wellness as they age: physically, mentally, emotionally, and spiritually.

The Group was, and continues to be, committed to sharing the generated knowledge for used by and for community members’ benefit [12, 13].

## Methods

The long history of Indigenous communities being subject to unethical behavior and approaches by researchers is well documented [13, 14]. Those approaches tended not to reflect Indigenous worldview and did not necessarily benefit Indigenous peoples or communities. As a result, Indigenous peoples continue to regard research, particularly research originating outside their communities, with a certain apprehension or mistrust [15].

The emerging Indigenous research paradigm in Canada calls for conducting research by and with (as opposed to on) Indigenous people [16]. This research used the community-based participatory research model and a strength-based approach to identifying strategies *Nehiyawak* women used to attain and maintain wellness as they age. Community-based participatory research is a collaborative approach that equitably involves all partners in the research process and recognizes the unique strengths that each brings [17]. This model is a philosophy and method that seeks to engage people and communities in all phases of research from the conceptualization of the research problem to the dissemination of the results [13, 18]. Community-based participatory research creates bridges between communities and researchers, through the use of shared knowledge and experiences, and it facilitates the establishment of mutual trust that enhances the quantity and quality of data collected. The key benefit of these collaborations is deeper understanding of a community’s unique circumstances, and a more accurate framework for adapting best practices to suit the community’s needs [13, 19]. A report from First Nations Information Governance Centre (FNIGC) [20] stresses the use of strengths-based approaches which focus on identifying and supporting the various strengths, motivations, ways of thinking and behaving, as well as the protective factors—within the person or the environment—that support people in their journeys toward well-being.

Indigenous community-based participatory research methods [10, 13] were used throughout this research. The seven members of the *Sohkitehew* Elders Advisory Committee were involved at each stage of the research process, to ensure the research was grounded in the community and in *Nehiyawak* tradition and culture.

### The roles of the Sohkitehew Group members

The *Sohkitehew* Group itself consists of members of the Maskwacîs community including staff of Maskwacîs Health Services, researchers from Maskwacîs and Universities of Calgary and Alberta, and the *Sohkitehew* Elders Advisory Committee.

The seven women Elders of the *Sohkitehew* Elders Advisory Committee provided the grounding and inspiration of the research. The Committee met regularly with Listener and Ross to discuss every aspect of the research, from initial idea, through the grant application stage, to the actual research, ensuring that the cultural aspects of the research were properly discussed and incorporated into the work. The Elders also attended and contributed to Sharing Circles and Community Workshops.

Two members of Maskwacîs Health Services staff, Graham (Director of Nursing) and Heckman (MD) were part of the research team, adding knowledge of the health and wellness issues impacting the community, and enabling the research findings to be widely disseminated in the community.

Four researchers transformed the research plans into practice in collaboration with other group members. Principal investigator (PI), Voyageur, was already well known in Maskwacîs and was involved in designing the research. She attended Sharing Circles, Group Meetings and events when possible and was important in disseminating the findings in a variety of settings. Investigators, Listener (research assistant) and Ross (co-PI), both had established relationships with the Elders in the *Sohkitehew* Elders Advisory Committee and other community members and implemented all aspects of the research in the Maskwacîs. Listener is a Maskwacîs community member from Ermineskin Band and was known by many of the research participants. Ross has worked in Maskwacîs on women’s health research since 2015. Oster (investigator) also had a longstanding relationship with Maskwacîs Health Services and community members, having developed and carried out several research studies in Maskwacîs. He was fully involved in designing and carrying out the research.

### Sharing circle methods

Community members were invited to join the Sharing Circles by displaying posters widely throughout the community. For example, they were posted on community notice boards, in the band offices and in the health centres. Public service notices were also broadcasted on the local community radio station, Hawk Radio. Community meeting notices were also included in Band newsletters [21]. The posters encouraged “*all community members, men and women of all ages*” to discuss aging, being healthy and living well. Anyone wishing to join the Sharing Circles was welcome to do so, including anyone who provided transport for a participant. We did not exclude anyone who wished to attend an event. Each participant signed an Informed Consent form before joining a Sharing Circle.

We chose to use Sharing Circles as our means of collecting information because they brought community members together to openly discuss aging well. Sharing Circles are a traditional communication method used by Indigenous people to discuss issues and topics in an egalitarian and supportive manner [22, 23]. Sharing Circles also provide a spiritual component to the research process and ensure the equality of all members of the Sharing Circle—including the facilitators [24]. This mode of communication also reflects the Indigenous people’s values of sharing, supporting each other and respecting life experiences through the use of personal interaction, and group consensus to identify problems and derive solutions [24]. Sharing Circles incorporate oral traditions and styles of interaction entrenched in the culture [24].

Sharing Circles were scheduled in easily accessible community spaces in different locations around the community so community members could attend the event closest to their home [21].

Two researchers were present at each Sharing Circle [25]. Listener, the project’s community-based research assistant, guided and facilitated the discussions. Ross supported the discussions and summarised the points made by participants. The two researchers supported each other’s roles during the Sharing Circles. Voyageur also joined when she was able to do so.

*Nehiwayak* tradition and culture were incorporated in a number of ways that were respectful to community members. The Sharing Circles opened with *Nehiyawak* ceremony, including Maskwacîs researcher, Listener, smudging the Sharing Circle meeting room to cleanse the space before the events. Listener also offered to smudge participants at the start of the meeting. Smudging is a cleansing ceremony that clears the mind, body, and heart of people who participated. Smudging involves placing dried sage leaves into a bowl and igniting the leaves with a match. As the flames are gently blown out, the smoke is wafted over each participant who wishes to take part. The person being smudged pulls the sage smoke towards them and gently inhales. This short ceremony helped prepare participants for the discussions that followed.

Elders from the *Sohkitehew* Elders Advisory Committee were invited using protocol (offering tobacco in the form of cigarettes) to provide welcoming prayers to start the meetings, and to bless and give thanks to Creator for the food. Committee Elders were able to attend all the events and they contributed guidance, cultural knowledge, and wisdom. Each Committee Elder who attended an event received an honorarium to acknowledge their valuable role.

Sharing food and visiting are important customs within the community. The Sharing Circles started in the morning with coffee and snacks. This gave the participants an opportunity to visit with their friends and catch up with community members before the morning discussion session. A healthy lunch was provided by a local caterer during a break when participants could have a more general discussion. The afternoon discussion was shorter and offered the opportunity to validate points and information offered by participants earlier in the day. The Sharing Circles closed with draws for prize draws. Each event lasted approximately 3 h.

The physical set-up of each meeting space had tables and chairs arranged in a circle with two easels standing in the open space. This meant the facilitator was easily seen by everyone. Seating was purposefully set out in a circle because this symbolized an important aspect of the *Nehiyawak* worldview. The circle represents the cycles of life with no beginning and no end. There is no time element and it symbolizes infinity and interconnectedness. When people come together in a circle, there is a spirit of oneness and sense of sacredness [26].

One of the two easels was used to display Jim Dumont’s description of the four aspects of the Medicine Wheel (spiritual, mental, physical and emotional) [7]. *Nehiyawak* and many other First Nations communities, commonly describe life as holistic and use the terms physical, mental (or intellectual), emotional and spiritual to describe their perception of health and wellbeing. Many communities have a tradition of using the Medicine Wheel to talk about and conceptualize health [6, 27]. One of the four wellness aspects was displayed at a time so the description could encourage the discussion. The other easel held the flip charts that were used to record the group members’ discussions.

### Data generation

The topic “aging well” was the focus of each Sharing Circle. The four wellness aspects of the Medicine Wheel (physical, mental, emotional and spiritual) were used as a basis for the discussions. Participants were greeted at the beginning and an Elder offered a welcoming prayer. The guidance for the Sharing Circle was described, including confidentiality of the discussions. Participants responded to this request by contributing their own experiences and strategies for attaining and maintaining wellness as they age. They also discussed what they had observed from others about aging well (for example family members). Discussions were planned as respectful interactions where all participants could contribute. Listener started the discussions by reading Jim Dumont’s wellness quote to provide the background for each discussion. The participants’ discussion points were recorded on the flip chart. Everyone present could contribute their own experience as they wished and could observe the information as it was being collected and add any additional comments, information or examples as needed.

This data collection method was designed to capture the Sharing Circle information during the actual discussions and in plain view. The written comments were captured on flip charts and participants could ensure accuracy and discuss or suggest additional points. The data collection method aimed to provide transparency in information gathering, to be true to the voices of the participants, and to reflect the shared understanding of the group.

We did not use a talking stick (or stone) because we were concerned this might inhibit discussion, since only the person holding the talking stick can speak. Audio recording was not used because this might have also inhibited discussion. For example, participants might have been concerned about what was being recorded or how their comments might be interpreted.

The data collection process ended after the third Sharing Circle since data saturation had been met. It was unlikely that any new insights, observations or themes would emerge from subsequent events.

Rigour of the Sharing Circle methods and data collection were incorporated in our study design in several ways. Firstly, our Sharing Circle method involved recording participants’ comments on a flip chart throughout each session, permitting open recording of all contributions, discussion and feedback from the participants. As well, Listener and Ross made their own personal notes immediately after the session to check that all important discussion points and the atmosphere of the meeting were captured. In addition, the researchers debriefed the day after each meeting specifically to review the notes from the flip charts and their own reflections about the meeting, to ensure that no important pieces of the discussions were omitted or misinterpreted. Voyageur and the Sohkitehew Elders were also available as necessary if Listener or Ross encountered any areas of concern.

### Data analysis

The flip chart information from all the Sharing Circles were transcribed. Document analysis of these data were conducted by qualitative researchers Alvadj (University of Alberta qualitative researcher) and Voyageur, using content analysis methodologies [28]. Analysis proceeded concurrently with data generation.

The data analysis group (Listener, Ross, Alvadj, and Voyageur) developed a preliminary content analysis framework built on the the Sharing Circle data, incorporating the ideas that emerged relating to the four aspects of the Medicine Wheel that helped guide each workshop. Further, this type of analysis enabled situating women in relation to women’s roles and their status in the family. Using this framework as the basis, content analysis was used to interrogate the notes of each Sharing Circle, and Listener and Ross’ personal notes and reflections about each session.

The first analysis of the data explored the participants’ perception of aging relating to the wellness aspects of the Medicine Wheel: physical, mental, emotional and spiritual wellness. As well, the ways women used to attain and maintain wellness in each aspect of the Medicine Wheel was explored. The detailed exploration of the data identified three themes related to wellness. The areas were: (1) culture/tradition, (2) self-care, and (3) family/intergenerational. Achieving balance between the wellness aspects was also addressed.

### Ethical considerations and consent to participate

The research was designed and undertaken according to community-based participatory research methods and Indigenous ways of knowing [10, 13, 19]. The OCAP Principles guided all aspects of the research, by involving community members in all stages of the research development and conduct, ensuring the community has access to the research and can determine how the findings will be used [29]. The research was developed with Maskwacîs community members and Maskwacîs Health Services representatives. The *Sohkitehew* Elders were involved in developing the research and supported the local dissemination of findings for the benefit of the people of Maskwacîs before considering publication. The *Sohkitehew* researchers had a research agreement with Maskwacîs Health Services and Maskwacîs Elders from 2017.

All methods were carried out in accordance with the relevant guidelines and regulations, including the Declaration of Helsinki. The research was approved by two Human Research Ethics Boards, University of Alberta (Pro00076772) and University of Calgary (REB17-0676). Both Boards adhere to Canadian ethical practices for research with Indigenous peoples: the OCAP Principles [29] and the Tri-Council Policy Statement (TCPS 2): Ethical Conduct for Research Involving Humans [30].

## Results

Three Sharing Circles were held in 2017 for participants to discuss the wellness strategies used to help them attain and maintain wellness as they aged [21]. The Sharing Circles were attended by 36 Maskwacîs community members (Table 1).

**Table 1:** Date and setting of events and attendance

Date	Setting	Attendees
10 April 2017	Recreation Center, large gym, easy access	23 (20 women, 3 men)
17 May 2017	Elders Center, comfortable medium-sized room, easy access	10 (9 women, 1 man)
26 June 2017	Large open hall, accessed by steps/ramp	8 (6 women, 2 men)

Sharing Circles were attended by 36 individual community members. Some Elders Advisory Committee members attended more than one of the sharing circles [21]

The participants at the Sharing Circles were open in discussing their perspectives about aging and wellness using their prior knowledge of the four aspects of the Medicine Wheel: physical, mental, emotional, and spiritual. They realistically discussed the topic of aging well, addressing both the positive and negative aspects associated with aging. All agreed that it was best to make the most of aging and to participate in activities that will keep them healthier and mobile for as long as possible. They believed that this was something that they could do for themselves and it was in their own hands.

### Physical wellness

#### Question asked: what does physical wellness mean to you?

According to participants, physical wellness means “*to be healthy and have mobility*” (SC1), remembering to “*keep active*” (SC2), and “*taking care of yourself so you feel good about yourself*” (SC2). Participants stressed their need to “*keep well*” (SC2) as they aged, both for themselves and for their families. Discussions of physical wellness also addressed the expected physical decline of the body as women age. Participants described aging-related concerns of losing hearing, sight, being wheelchair-bound, skeleton deteriorating, losing their independence and apprehension about the unknown impacts they may experience as they age. Sharing Circle participants felt it would be good to know how to prevent aging, but acknowledged it was not possible, and agreed that there were ways to make the most of aging for example by eating well and enjoying the activities they can do.

#### Maintaining physical wellness

One woman talked about her 96-year-old *Mosom* (Grandfather), who taught her throughout her life – he worked hard all his life, attending to his job, yard, house. Now he works mainly around the house to stay full of life. He has taught and modelled an active life. This tells his granddaughter that he believed the key to remaining healthy and well is to keep active (SC2). Participants discussed preventative steps to staying well and this included listening to your body. This means being aware of any changes to the body or how you feel, seeking help if needed, approaching a Doctor or other individual who can provide help.

Traditional/cultural practices were described to maintain physical wellness, such as attending sweat lodges, taking part in round dancing and hand games, and doing crafts such as beading. However, self-care was stressed as being particularly important, including physical activities, eating a healthy diet, having good sleep habits, drinking plenty of water and doing lots of walking. Rest was also considered important – for example the luxury of “*Kokom (Grandmother) naps*” (SC1). Recommended self-care physical activities varied widely; in addition to walking, participants mentioned swimming, gardening, continuing to work around the house. Participants felt it is important to choose activities you enjoy so that you do not see them as a burden.

#### Family/intergenerational activities

Participants felt urged to pass on physical wellness strategies to their relatives. There was particular concern for the unhealthy choices being made by some family members that were deemed to be damaging. They believed that young women need to learn how to make better choices to maintain their health by avoiding harmful foods, drugs and alcohol. “*Young women need to know what not to put in their bodies, because they cause harm*” (SC1). As an example, they suggested that young women should choose healthy snacks, such as “*pineapple versus cheeseburgers*” (SC1).

There is a need for sharing information about wellness issues associated with different stages of a woman’s life. For example, the pre-pubescent girls must be taught about the changes that occur to their body as they reach puberty and about menstruation. Some participants mentioned the traditional teachings given to young girls as they move from childhood to womanhood. One commented, “*girls need to know about these changes to stop them being anxious or frightened*” of these physical changes (SC1). Teenage girls must learn how to respect their bodies, how to dress and behave, and also about sexual health. Family planning, contraceptives and healthy sexuality are topics that are not easily talked about but are necessary to allow young people to make healthy choices.

Further, as women age, they encounter many bodily changes associated with menopause. All of the topics mentioned above must be frankly and respectfully spoken about to ensure that individuals have the information that they need to determine whether changes they are experiencing are normal and if they need to seek medical care for any abnormalities. One participant stated that she did not have anyone to talk to about menopause because her mother has Alzheimer’s (SC3).

### Mental wellness

#### Question asked: what does mental wellness mean to you?

According to participants, achieving mental wellness included being calm and happy, by thinking positive thoughts, staying healthy, having a good memory, and resting. Apprehension was expressed several times about future loss of memory or developing an illness such as Alzheimer’s disease or dementia which would impact their mental capacity and lessen their ability to take care of themselves. In association with this topic, there was also concern that their perceptions of themselves, their world and their loved ones would change. They worried their family member would no longer be themselves, and perhaps, they would not know their own family members.

#### Maintaining mental wellness

Participants mentioned the need for mental health self-care begins with the individual. They commented that obtaining good mental health starts with a need for honesty and openness so the person can openly admit that there is a problem. There must be an awareness and acknowledgement of a problem before actions can be taken to work towards a solution.

To maintain mental wellness, it is important to “*be mindful and present, sometimes the answer is right in front of you*” (SC3). One participant stated that “*providing yourself with positive affirmation is VERY important. You need to look in the mirror and tell yourself that you’re stronger than you think you are. Other people don’t often say positive things so you need to provide that input yourself”* (SC2). Participants advised, “*Be your best supporter, believe in yourself and that you can be that way*” (SC2), “*Think about your own self-care first – think positively! and think happy thoughts”* (SC1), further, “*don’t be hard on yourself, be kind*” (SC2) and “*allow yourself to make mistakes, no one is perfect*” (SC2)*.* It is also important to apologize for past damaging events for which you feel responsible. That apology is “*for you, not for them*”, allowing you to let that action go and move forward (SC3).

Participants discussed attaining and maintain mental wellness by taking part in traditional/cultural activities such as feasts, sweat lodges, round dancing, pow wows, hand games, and other cultural gatherings; creating traditional arts and crafts was also suggested. Non-traditional practices such as doing jigsaw puzzles, playing games, going to movies, yoga and imagery were also suggested as ways to help maintain mental wellness. “*It is important to keep busy (avoid getting lazy), but know when to stop and ask for help*” (SC2).

#### Family/intergenerational activities

Some women stressed the need to keep learning as they aged and felt grandchildren can be a help in this regard, for example by teaching computer skills and games, “*to keep your brain active*” (SC2). They discussed the mental changes they had noticed as they aged, and the importance of continuing to learn new skills to nourish the mind. Another suggested technique was to “*disconnect with ‘life’ and turn off technology for a day*” (SC3).

### Emotional wellness

#### Question asked: what does emotional wellness mean?

Emotional wellness was said to be “*about feelings*” (SC2), understanding and expressing emotion. Participants agreed that emotional wellness involves being empathetic, compassionate and kind to self and others. It can be hard to be emotionally well, particularly if individuals are unable to help themselves in a particular situation. For example, people may be troubled about the past and be in a “*state of trauma*” (SC3). Not everyone has learned how to manage their emotions: “*Maybe they don’t know the steps. They need to be provided with guidance and empowerment*” (SC3).

#### Maintaining emotional wellness

Self-care and self-acceptance are both very important to maintain emotional wellness. It is necessary to “*understand your emotions*” (SC2), and to experience the full spectrum of emotions: “*laugh, cry, be happy, let yourself experience and release these emotions*” (SC2). To avoid being stuck with emotions that cannot be defined, *“you need to work at being positive, so that you feel good about yourself*” (SC2). Specifically, it is important not to “*hold on to anger, it hurts you—let it go and be free*” (SC3).

Grief was mentioned as a specific emotional issue: “*Young women need to know about grief and how to deal with it, particularly in this community. They need to know that grief is normal, and that they are not alone in dealing with grief*.” (SC1). Another participant said, “*Don’t forget to deal with grief! If you don’t deal with it, it will deal with you. It will make you sick.*” (SC3).

It is very important to be in tune with others’ emotions, to be empathetic to other people, although “*it’s hard to be compassionate and kind, it doesn’t necessarily come easily – there are rude and difficult people, and it’s hard to be nice to them*” (SC3). Participants stressed that everyone has problems, but they need to find their own ways to deal with their individual issues.

It is also very important to express gratefulness and joy: “*always be in a state of gratefulness even for small things, life is full of gifts, there’s always something to be grateful for*” (SC3). Traditional and cultural activities were also important to support emotional wellness, including round dancing, crafts such as beading and sewing, and traditional cooking were also described as being useful. Other self-care activities included taking exercise, drawing or coloring.

#### Family/intergenerational activities

Participants believed that childhood is the best time to learn the important skills to deal with emotional wellness: “*It is particularly hard for kids to understand their emotions – they need to be taught about their emotions and how to cope with them”* (SC2*)*. One *kokum* talked about a grandchild who couldn’t explain what she was feeling, she stood stiff with hands clenched describing her feelings as “*Rrrrrr*!” but could not find words to elaborate (SC2). Communicating love between family members can help to teach children to love themselves and others:

*“Tell your children you love them and hug them. Tell them good morning. Tell them how much they mean to you. Parents may not always say I love you, but they will show you they love you.”* (SC3)

### Spiritual wellness

#### Question asked: what does spiritual wellness mean?

“*Spiritual wellness is the core of being—the mind is well*” (SC1). Sharing Circle participants discussed having a belief in a higher power, and the need for individuals to take care of their own soul and beliefs. Spiritual wellness was part of daily living. One woman Elder said *“Elders are meant to know everything – we don’t! We do know some things about spirituality.”* (SC2). She explained that she had been in Residential School and did not learn her traditional and cultural ways. She tries to learn as much as she can about culture and shares that knowledge with her grandchildren so that they have better awareness than she did. Other women shared similar experiences of residential schools and colonization and reiterated the importance of sharing spiritual knowledge and experiences with family members.

#### Maintaining spiritual wellness

Participants discussed holding the belief that “*everything happens for a reason, it’s up to you to find the good*” (SC3) is an important way to maintain spiritual wellness. Women described many cultural/traditional activities in relation to spiritual wellness, in particular taking part in “*cultural ceremonies—a constant reminder of unconditional love*” (SC1). Specific examples of spiritual activities were feasts, round dancing, sweat lodges, hand games, smudging, praying to the Creator, and attending Sundance ceremonies. Less traditional or ceremonial self-care activities were important in women’s everyday lives; these activities included meditation, yoga, prayer, attending church, using affirmations, and maintaining a safe place. Women believed they must “*Take care of your own self, take time for yourself, smudge as part of self-care*” (SC2). “*You must listen to your intuition, trust in yourself*” (SC2): if something seems wrong, it probably is and should be avoided. They should be strong, building on childhood experiences “*Laugh, dance in the rain, play, be fearless. Do all the things you liked to do as a kid. Remember the joy that kids have. They just do! See the joy in the little things.*” (SC3).

#### Family/intergenerational activities

The participants believed that “*traditional values need to be passed on to kids from Elders to maintain the traditional ways. For example, prayers can be said anywhere*” (SC3) That is, prayer does not need to be restricted to specific locations. Several participants believed that “*smudging needs to be taught in the community”* (SC3) because many community members have not received that teaching from their own families. The importance of smudging was mentioned in all Sharing Circle discussions.

### Perception of balance to achieve wellness

**Fig. 1:**
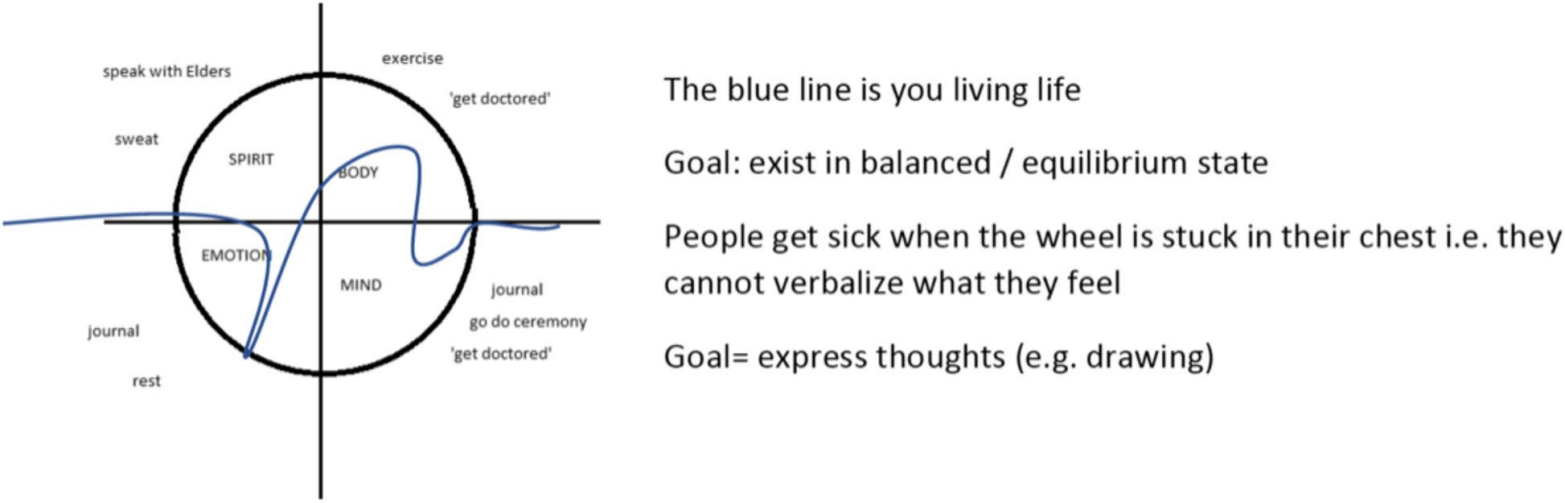
Participant’s personal description of balance

The concept of balance emerged organically during the third Sharing Circle. The participants were acquainted with and discussed the Medicine Wheel’s four aspects of wellness related to aging, and also spent time discussing balance between the four aspects – if the four aspects are unequal then a person cannot achieve well-being. This final Sharing Circle started out with three participants, with other participants joining throughout the meeting. With this session starting as a small group and gradually expanding, the session had a more intimate feeling. One participant described in detail her views about maintaining balance in life. This participant created a diagram illustrating her personal description of balance (Fig. 1).

She explained the figure as follows:

*There are four areas of concern, spiritual, emotional, mental and physical. You need to identify that you’re out of balance, and if so, which area needs attention. If you’re out of balance in any of the four areas, you need to deal with that specifically. To do that you need to be mindful and present, to find where the energy dips are and need to deal with those imbalances – these need to be “doctored”* (SC3).

This participant discussed paying special attention to balancing the different Medicine Wheel aspects as she was drawing the diagram. In particular, she highlighted the need to be mindful and present to be able to identify and address any imbalances.

Other participants also discussed needing to “*have equilibrium*. *The body cries out if we don’t have balance. You need to remember to be mindful and present to see when the balance is off*” (SC3). “*Have the balance—when we don’t, we start to have sickness*” (SC3).

It was important for participants to have “*a holistic approach to life*” (SC3), and to teach that way of life to grandchildren.

### Results summary

**Fig. 2:**
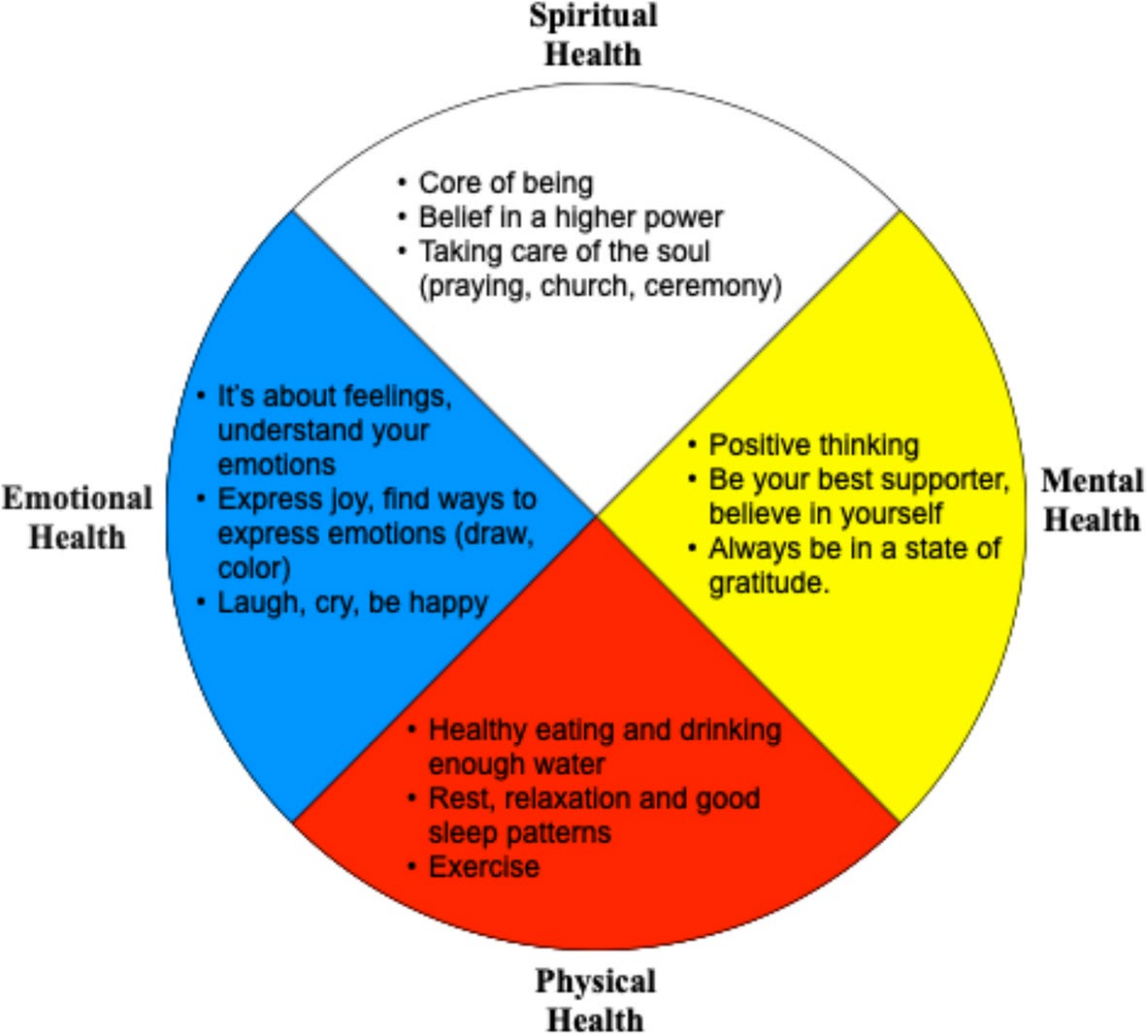
Depiction of the Medicine Wheel, including women’s strategies to maintain wellness as they age

The Sharing Circles used the Medicine Wheel as the framework to explore participants’ ways of aging well. The results identified practical strategies for supporting aging well in each area of the Medicine Wheel: physical, mental, emotional, and spiritual (Fig. 2). These useful strategies were connected with enhancing culture/tradition, self-care, and family/intergenerational aspects of life.

## Knowledge dissemination

Knowledge translation in Maskwacîs was an essential part of our research planning, with the intention to first share the research findings with the women of Maskwacîs [12, 13]. Working with the Elders, the first stage was planned to present the research findings at feedback sessions in a variety of community locations to seek further feedback on the results. We then planned to develop and distribute booklets that provided the information for easy access in the community. We had found these methods very successful in distributing our earlier menopause research findings in and around Maskwacîs [11].

### Community feedback

Three community feedback sessions were held to directly present the findings from the Sharing Circles to the wider community between April 2019 and January 2020. These informal workshops were attended by *Sohkitehew* Research Group members and Elders when they were available, and incorporated lunches and prize draws. The sessions were attended by women from across Maskwacîs, and participants discussed the findings in the context of their own lives and families. They stressed the need for information about successful wellness strategies to be made available for the community more widely and discussed a variety of options including further workshops, written materials (for example pamphlets or booklets) that could be freely available in the community.

### Development of a booklet about strategies for aging well

Following the community feedback sessions, the *Sohkitehew* Elders Advisory Committee and the research team identified key practical strategies for aging well that were found useful by the Sharing Circle participants. These strategies were discussed and refined during several *Sohkitehew* Elders Advisory Committee meetings. Together with the *Sohkitehew* Elders, the research team designed aging well booklets based on the results of the Sharing Circles. Once finalized these were formatted into a draft booklet which incorporated archive photographs of Maskwacîs women and families from the Samson Cree Nation Museum and Archives.

The *Sohkitehew* Research Group held meetings in three of the communities that make up Maskwacîs to present and discuss the draft booklets, and incorporated the suggestions from the community in the current version.

### Impact of the COVID-19 pandemic

**Fig. 3:**
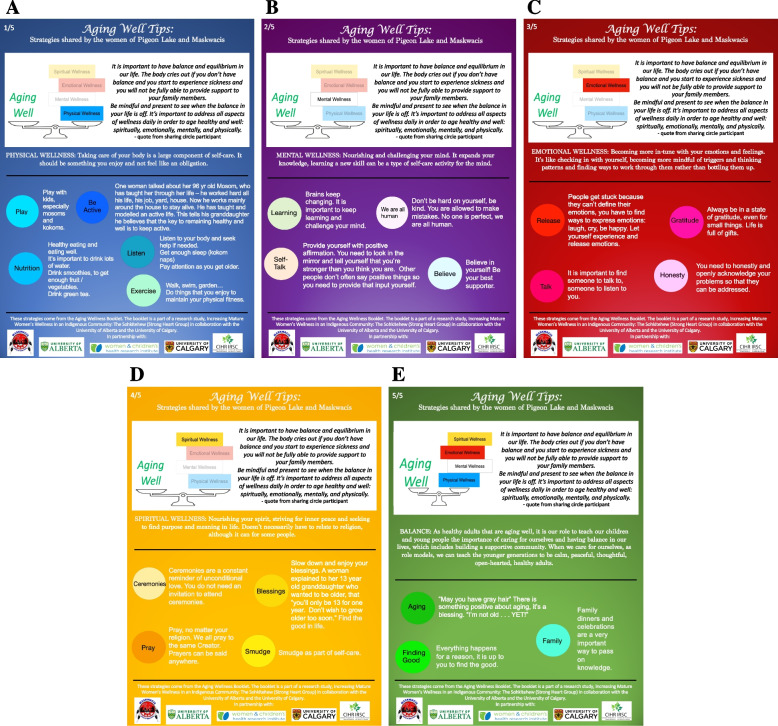
Aging well cards

We were about to distribute the booklet in Maskwacîs in early 2020, just as the COVID-19 pandemic began in Alberta. Our plan had been to hold a community launch, and to distribute booklets through Maskwacîs Health Services, however the ongoing pandemic prevented us from holding further community feedback sessions or distributing the booklets in the community. In discussion with the *Sohkitehew* Elders Advisory Committee we developed brief posters, now freely available through public access on the Maskwacîs Health Services website (Fig. 3).

We have continued to revise the aging well booklets and are currently developing a *Nehiyawak* version which will including translation into syllabics and Cree roman orthography. We are also considering additional media opportunities such as Band newsletters and Facebook.

Although the distribution of the information developed through this research was hampered by the COVID-19 pandemic, we maintained contact with the *Sohkitehew* Elders Advisory Committee and other community members who remain enthusiastic about making the findings of the research widely available. We are committed to making the information about aging well widely available in the community.

## Discussion

This preliminary community-based participatory research enabled the *Sohkitehew* Research Group to bring Maskwacîs community members together to develop a mutual understanding about older women’s lived experiences of “aging well” through open discussion, using the Indigenous Medicine Wheel as our guide. The Sharing Circles identified valuable knowledge and understanding that led to the generation of information respecting and honouring Indigenous ways of knowing to help other women in the community as they age. The information has been presented widely and discussed in Maskwacîs to seek further community feedback. The practical strategies for aging well have been developed in a variety of formats, such as booklets and online information, so that women and their families can easily access advice about aging well.

### Aging well

Participants identified and discussed the ways they themselves used to attain and maintain wellness as they age, sharing their own strategies with the other participants. They were comfortable discussing the topic of aging, in a realistic and balanced way, addressing the positives and benefits alongside the challenges and limitations related to aging.

Each of the Sharing Circles highlighted the importance of family and relations to aging well, identifying ways that family members can support each other. Traditional and cultural activities are vital in attaining and maintaining wellness and protecting the *Nehiyawak* way of life. These activities varied widely, from traditional cooking and crafts, gatherings such as pow wows and feasts, dancing, and collecting therapeutic plant roots and leaves for medicines.

The participants believed that it was important for them to demonstrate and teach the value of their traditional ways in their families, although they felt many younger family members were not yet ready for these ideas. Indeed, several of the participants had themselves come to traditional ways later in adulthood, meaning that they understood clearly that they needed to make sure that their families had opportunities to learn.

Other researchers have also highlighted the importance of intergenerational communication and social activities for older people to maintain wellness, for example nurturing intergenerational social engagements [31], stressing the benefit Elders experience from spending time with children [32]. These activities are available within families, but there is also a need to incorporate intergenerational programs in communities for people whose families have been disrupted through colonization [1, 2]. Bringing generations together may be difficult to achieve because younger and older people often feel more comfortable in the company of their own age groups [31].

### Research design

Several aspects of our research design are worth highlighting. Most importantly, the research design was developed as a collaboration between the researchers and the *Sohkitehew* Elders Advisory Committee, incorporating collective decision-making [33] and including several *Nehiyawak* features.

#### Importance of integrated knowledge translation

Integrated knowledge translation is vital, particularly in community-based participatory research. Building on our previous research [11], we developed a knowledge translation plan that would involve ongoing collaboration between community members, Elders and researchers to design and carry out the research and plan how the findings would be disseminated, using the results of this study directly to benefit the community. This comprehensive approach is seldom seen in practice [13]. Our dissemination plan involved taking the results directly to the community through larger community events. The continuing effort has been hampered by COVID-19, which prevented the community coming together over the past two years, however we intend to continue this work when COVID-19 restrictions permit.

The results of this research are important because aging is not a topic that is often discussed, except among older people: yet aging will affect everyone. As indicated from the Sharing Circle discussions, the people of Maskwacîs believe that it is particularly important to communicate ways for younger women to remain healthy and well as they age.

#### Medicine wheel

Our use of the Medicine Wheel as the framework for the *Sohkitehew* Sharing Circles was a significant strength of the research, because the participants were comfortable to talk about the *Nehiyawak* Medicine Wheel teachings that are part of their everyday lives, particularly the four aspects of “human nature”, that is physical, mental, emotional, and spiritual [34]. Participants demonstrated their familiarity with the Medicine Wheel and highlighted the importance of working towards achieving balance among the four aspects to achieve wellness for themselves. They acknowledged that achieving personal balance required effort as described by Gesink [6]. They also stressed the need to first achieve personal balance before being able to help others [6]. Other researchers have noted that factors outside individuals’ control can adversely impact health and wellbeing, such as societal, historic and political factors [35]. These external factors can lead to lack of economic security, an area where Indigenous women specifically need support [6].

The Medicine Wheel also guided our interview analysis and presentation of the study results, allowing us to use this structure to bring together the contributions of participants in each of the three sharing circles, thus providing a rich description of the data.

#### Sharing Circles (data generation)

Our research used Sharing Circles to generate the research data, another strength of our method. The details of our Sharing Circles differed from those described by others, such as Rothe and colleagues [36] who explored risk taking and injury prevention in a Canadian First Nation, and Tachine and colleagues [37] who carried out research with University of Arizona Native students. Our Sharing Circles were adapted to be appropriate for the context of our wellness research in Maskwacîs, however the principles are the same—paying careful attention to cultural protocol and using an open-structured, conversational style for the sessions. This method was appropriate for our study, because the Sharing Circles often involved discussion between several participants who knew each other, so that they could share their experiences in a more organic way than in a more structured talking circle [22].

### Limitations and strengths

Our study has several limitations. The research was carried out in one Indigenous community, and the proposed strategies may not apply directly elsewhere, however our methods could be applied elsewhere to generate community-specific findings and output. Our Sharing Circles were carried out during the day: this obviously suited some community members, however may have limited the number and type of people who were available to contribute. We consciously decided not to collect personal information such as age because this might be considered invasive: in fact, this might have been an overly protective decision, because most individuals knew the other people joining the Sharing Circle. The presence of men at the sessions may have meant that some important topics were not discussed. To maintain confidentiality our data collection method did not attribute specific comments to individual participants. As well, topics were discussed openly, so some comments resulted from several contributions, and perhaps reflect an aspirational view of aging well rather than one can be attained by everyone.

The strengths of the research are that the ideas were generated by community members themselves. The strategies are both practical and suitable for daily use, empowering women to consider healthy choices as they age. The participants’ life experiences were mixed, and yet there is a cohesiveness about their combined suggestions for aging well. This is in part because their recommendations are based on open discussion between community members during the Sharing Circles, where it was possible to reach a shared outcome. As well, our method was influenced by our use of the Medicine Wheel [7], which was well-known to the participants, and it seems they already turn to strategies suggested by the Medicine Wheel, including working to achieve balance between the four aspects of physical, mental, emotional, and spiritual wellness.

The involvement of the *Sohkitehew* Elders Advisory Committee was vital to the success of this study. The Elders’ individual and collective input helped to ensure that each aspect of the research remained true to *Nehiyawak* tradition and culture, adhering to the values and practices of the community.

The knowledge generated by our research lends itself well to wide distribution and use in Maskwacîs, which was our original goal. The development of booklets offering advice (from the research participants) directly to community members will allow for easy distribution of accessible information. Our low-technology methods would also be amenable to translation to other more modern media approaches, such as online versions of the information and advice that may be made available more widely outside Maskwacîs.

## Conclusions

The goals of this initial project were to collect valuable strengths-based knowledge to generate information respecting and honouring Indigenous ways of knowing to help other women in the community to achieve wellness as they age. Using Sharing Circle methods, *Nehiyawak* participants discussed physical, mental, emotional, and spiritual ways to attain and maintain wellness as they age, based on community values and beliefs. The *Sohkitehew* Elders and other community members were involved at all stages in the research, to ensure the work was grounded in the community and in *Nehiyawak* tradition and culture. Through this community-based participatory research, Maskwacîs community members identified simple, practical suggestions for aging well that can be applied in everyday life.

This study utilized a strength-based perspective to raise awareness about women’s personal strategies for aging well, rather than viewing aging as a deficit. The ideas generated by Maskwacîs community members are wellness strategies that are practical, suitable and accessible suggestions that the women themselves found helpful and can be adopted into daily life, empowering women to consider healthy choices and use their own strengths to age well. Aging is not a topic that is often discussed, except among older people: yet it is something that everyone will experience.

Although developed with and for the Maskwacîs community, the results of this important research would be relevant and useful for many people in other Indigenous communities with similar issues and may also prove valuable for women in non-Indigenous settings. Other people can use these methods and findings as a basis to talk about aging well with their own communities, helping to raise awareness about women’s concerns related to healthy aging.

Our research may also form the basis of wellness research more widely because our work is based on trusting relationships between researchers and the community, with the goal to first benefit the community itself.

This Sharing Circle study also set the scene for the next stage of our research which uses in-depth qualitative one-to-one interviews with individual *Nehiyawak* women. The goals of that research are to learn more about the practical aspects of aging well, to help women to find their own cultural ways to move towards wellness, and to make this knowledge available in the Maskwacîs and to other Indigenous communities.

## Data Availability

The data generated or analyzed during this study are available from the corresponding author on reasonable request.

## Abbreviations

MHS: Maskwacîs Health Services
MESC: Maskwacîs Education Schools Commission
MCC: Maskwacîs Cultural College
PI: Principal Investigator
OCAP: First Nations principles of ownership, control, access and possession
TCPS-2: The Tri-Council Policy Statement: Ethical Conduct for Research Involving Humans
CBPR: Community based participatory research

## Footnotes

1 Indigenous people lived for thousands of years on lands that became Canada. Indigenous people now living in Canada include Inuit people, living mainly in the far North of Canada, and Metis people, descended from mixed European and Indigenous ancestry. “First Nation” is the term used to describe Indigenous peoples who hold reserve lands in Canada, including the *Nehiyawak* people of Maskwacîs, Alberta.

## References

[1] Gracey M, King M (2009). Indigenous health part 1: determinants and disease patterns. Lancet.

[2] Wilson K. Therapeutic landscapes and First Nations peoples: an exploration of culture, health and place. Health Place. 2003;9:83–93. 10.1016/s1353-8292(02)00016-3 12753791

[3] Oster R, Grier A, Lightning R, Mayan M, Toth E. Cultural continuity, traditional indigenous language, and diabetes in Alberta First Nations: a mixed methods study. Int J Equity Health. 2014;19(13):92. 10.1186/s12939-014-0092-4. 10.1186/s12939-014-0092-4 PMC4210509 25326227

[4] Viscogliosi C, Asselin H, Basile S, Borwick K, Couturier Y, Drolet MJ, Gagnon D, Obradovic N, Torrie J, Zhou D, Levasseur M. Importance of indigenous elders’ contributions to individual and community wellness: results from a scoping review on social participation and intergenerational solidarity. 2020;111(5):667-681. 10.17269/s41997-019-00292-3. 10.17269/s41997-019-00292-3 PMC7501322 32109314

[5] Halseth R (2013). Aboriginal women in Canada: gender, socio-economic determinants of health, and initiatives to close the wellness-gap.

[6] Gesink D, Whiskeyjack L, Guimond T (2019). Perspectives on restoring health shared by Cree women. Health Promot Int.

[7] Dell CA, Dell D, Dumont T, Fornssler B, Hall L, Hopkins C. Connecting with culture: growing our wellness. Facilitators’ Handbook. Saskatoon: University of Saskatchewan, Research Chair in Substance Abuse; 2015. http://www.addictionresearchchair.ca/creating-knowledge/national/honouring-our-strengths-culture-as-intervention/growing-wellness-connecting-with-culture/. Accessed 7 June 2022.

[8] Graham H, Martin S (2016). Narrative descriptions of miyo-mahcihoyān (physical, emotional, mental, and spiritual well-being) from a contemporary néhiyawak (Plains Cree) perspective. Int J Ment Health Syst.

[9] Richmond C, Ross N (2009). The determinants of first nation and Inuit health: a critical population health approach. Health Place.

[10] Wilson S. Research is ceremony: indigenous research methods. Winnipeg: Fernwood Publishing; 2008.

[11] Sydora BC, Graham B, Oster RT, Ross S. Menopause experience in First Nations women and initiatives for menopause symptom awareness; a community-based participatory research approach. BMC Womens Health. 2021;21(1):179. 10.1186/s12905-021-01303-7. 10.1186/s12905-021-01303-7 PMC8077762 33902542

[12] Jacklin K, Kinoshameg P. Developing a participatory Aboriginal health research project: “Only if it’s going to mean something.” J Empir Res Hum Res Ethics. 2008;3(2):53–67. 10.1525/jer.2008.3.2.53. 10.1525/jer.2008.3.2.53 19385745

[13] Jull J, Ninomiya M, Compton I, Picard A (2018). Fostering the conduct of ethical and. Equitable research practices: the imperative for integrated knowledge translation in research conduted by and with indigenous community members. Res Involv Engagem.

[14] Smith L (1999). Decolonizing methodologies: research and indigenous peoples.

[15] Canadian Institutes of Health Research, Natural Sciences and Engineering Research Council of Canada, and Social Sciences and Humanities Research Council. Tri-council policy statement: ethical conduct for research involving humans. 2018. https://ethics.gc.ca/eng/policy-politique_tcps2-eptc2_2018.html. Accessed 7 June 2022.

[16] McGregor D, Bayha W, Simmons D (2010). “Our responsibility to keep the land alive”: voices of Northern indigenous researchers. Pimatisiwin.

[17] Baydala L, Ruttan L, Starkes J (2015). Community-based participatory research with aboriginal children and their communities: research principles, practice and the social determinants of health. First Peoples Child Fam Rev.

[18] Fletcher C (2003). Community-based participatory research relationships with aboriginal communities of Canada: an overview of context and process. Pimatisiwin.

[19] Gabel C, Pace J, Ryan C (2016). Using photovoice to understand intergenerational influences of health and well-being in a Southern Labrador Inuit Community. Int J Indig Health.

[20] First Nations Information Governance Centre (FNIGC). Strengths-based approaches to indigenous research and the development of well-being indicators. Ottawa; 2020. https://fnigc.ca/wp-content/uploads/2021/05/FNIGC-Research-Series-SBA_v04.pdf.

[21] Khetarpal N, Listener L, Oster R, Ross S, Voyageur C (2021). Implementing community-engaged participatory research methods in a study of Cree women’s wellness: describing recruitment process and outcomes. Spectrum.

[22] Lavallée LF (2009). Practical application of an indigen research framework and two qualitative indigenous research methods: sharing circles and anishnaabe symbol-based reflection. Int J Qual Methods.

[23] Waddell CM, Herron RV, Gobeil J, Tacan F, De Jager M, Allan JA, Roger K (2020). Grounded in culture: reflections on sitting outside the circle in community-based research with indigenous men. Qual Health Res.

[24] Waddell CM, de Jager MD, Gobiel J, Tacan F, Herron RV, Allan JA, Roger K (2021). Healing journeys: indigenous men’s reflections on resources and barriers to mental wellness. Soc Sci Med.

[25] Bull JR (2010). Research with aboriginal peoples: authentic relationships as a precursor to ethical research. J Empir Res Hum Res Ethics.

[26] Hill DL (2006). Sense of belonging as connectedness, American Indian worldview, and mental health. Arch Psychiatr Nurs.

[27] Bartlett JG (2005). Health and well-being for Métis women in Manitoba. Can J Public Health.

[28] Mayan MJ. Essentials of qualitative inquiry. New York: Taylor & Francis Group; 2009. ISBN: 978159874107.

[29] First Nations Information Governance Centre (FNIGC). Ownership, Control, Access and Possession (OCAP™): the path to First Nations information governance. Akwesasne QC & Ottawa ON: First Nations Information Governance Centre (FNIGC); 2014.

[30] Canadian Institutes of Health Research, Natural Sciences and Engineering Research Council of Canada, and Social Sciences and Humanities Research Council of Canada. Tri-Council Policy Statement (TCPS 2): ethical conduct for research involving humans. 2018.

[31] Cornect-Benoit A, Pitawanakwat K, Walker J, Manitowabi D, Jacklin K, Wiikwemkoong Unceded Territory Collaborating First Nation Community (2020). Nurturing meaningful intergenerational social engagements to support healthy brain aging for Anishnaabe older adults. Can J Aging.

[32] Baron M, Fletcher C, Riva M (2020). Aging, health and place from the perspective of elders in an Inuit community. J Cross Cult Gerontol.

[33] Loppie C (2007). Learning from the grandmothers: incorporating indigenous principles into qualitative research. Qual Health Res.

[34] Rieger K, Bennett M, Martin D, Hack TF, Cook L, Hornan B. Digital torytelling as a patient engagement and research approach with First Nations women: how the medicine wheel guided our Debwewin journey. Qual Health Res. 2021;31(12):2163–75. 10.1177/10497323211027529. 10.1177/10497323211027529 PMC8564217 34238067

[35] Graham H, Stamler LL. Contemporary perceptions of health from an indigenous (Plains Cree) perspective. Int J Indigenous Health. 2010;6(1). 10.18357/ijih61201012341.

[36] Rothe JP, Ozegovic D, Carroll LJ. Innovation in qualitative interviews: “Sharing circles” in first nation community. Inj Prev. 2009;15:344–340. 10.1136/ip.2008.021261. 10.1136/ip.2008.021261 19805603

[37] Tachine AR, Yellow Bird E, Cabrera NL (2016). Sharing circles. Indigenous knowledge as a mode of inquiry (special issue). Int Rev Qual Res.

